# Gaucher disease in Iraqi children (Clinical, diagnostic & therapeutic aspects)

**DOI:** 10.12669/pjms.322.9316

**Published:** 2016

**Authors:** Rabab Farhan Thejeal, Ausama Jamal Kadhum

**Affiliations:** 1 Dr. RababFarhanThejeal, C.A.B.P. Assistant Professor, Department of pediatrics, Child Welfare Teaching hospital, Baghdad, Iraq; 2 Ausama Jamal Kadhum, M.B.Ch.B. Department of pediatrics, Child Welfare Teaching hospital, Baghdad, Iraq

**Keywords:** Gaucherdisease, Enzyme replacement therapy, Cerezyme

## Abstract

**Background and Objective:**

Gaucher disease is the most common inherited lysosomal storage disorder. It is a multi organ disease affecting bone marrow, liver, spleen, lungs, and other organs contributes to pancytopenia and massive hepatosplenomegaly. This study aimed to spotlight on clinical and laboratory characteristics of children with Gaucher disease to raise awareness among physicians about the disease and to evaluate the outcome of enzyme replacement therapy (ERT).

**Methods:**

Clinical courses were reviewed in 30 patients with age (2-22 years) with Gaucher disease. After starting (ERT), assessment of response included serial measurements of hematological parameters, spleen and liver sizes, symptoms and signs of bone disease, growth and severity scores were also evaluated.

**Results:**

The most presenting age group was (1 – 5) years (60%). Abdominal distension was the most common presenting symptom, Splenomegaly presented in all of the patients. A significant response to ERT was observed, weight and height increased, both liver and spleen sizes decreased. Hemoglobin level normalizedin (67%) of the anemic patients, platelet count normalized in (53.8%)after 6 months from (ERT), the mean of severity scoring index decreased with ERT from (10.2±5.8) to (7.8±5.7) after one year of treatment.

**Conclusion:**

Using ERT was safe and effective in the reversal of hematological complications and organomegaly in most of the patients.

## INTRODUCTION

Gaucher disease (GD), the most common inherited lysosomal storage disorder, is a multiorgan disease due to an autosomal recessive defect of the gene encoding glucocerebrosidase enzyme.^1^ It is not that rare in Iraq, exact incidence is hard to find out according to the troubled war situations, but according to (right diagnosis from healthgrades)^2^ it is around 1/50000GD is divided into two major types; neuronopathic & non-neuronopathic disease.

### Type 1 Gaucher disease

Type 1 Gaucher disease has a particularly wide variation in clinical signs, symptoms and disease course. In some cases, symptoms may begin in childhood and rapidly worsen over time. In other cases, the first symptoms may only be noticed well into adulthood.^3^,^4^

Perhaps the most common sign of Type 1 Gaucher disease is an enlargement of the spleen and/or liver. Patient presented with abdominal distension and may be huge. Overactivity of the enlarged spleen may result in an increased tendancy for bleeding, epistaxis, mouth or gum bleeding due to decreased platelets, or fatigue related to anaemia. Some patients undergo splenectomy because of splenic enlargement that interferes with their life activity.^5^

Cytopenia develops in patients who have undergone splenectomy reflects advanced marrow infiltration by Gaucher cells.

Other findings of GD involvement of the liver are hepatomegaly. Which occur in about 50% of patients.^5^ skeletal pathology in Gaucher disease is multifaceted. Three often coexistent pathologic presentations have been identified: focal disease (such as lytic or sclerotic lesions), local disease (such as Erlenmeyer (EM) flask shape deformity) and generalized osteopenia and osteoporosis.^6^

### Neuronopathic Gaucher Disease

### Type 2 GD

Type 2 Gaucher disease is a very rare, rapidly progressive form of the disorder that affects the brain as well as the organs affected by Type 1 GD. It is characterized by severe neurological involvement in the first year of life. Infants typically appear normal during the first few months of life, but would not survive more than two years, due to pneumonia or uncontrolled neurological deterioration that consequently leads to respiratory failure.^7^

### Type 3 GD

Type 3 is characterized by a slowly progressive brain involvement, in addition to serious involvement of other organs, also it is very rare. The signs and symptoms of this type appear in early childhood similar to those of type 1, so patients may be diagnosed as type 1 before central nervous system involvement.^8^

### Diagnosis

The “gold-standard” method for the diagnosis is finding deficient activity of acid β-glucosidase activity in nucleated cells, peripheral leukocytes, cultured fibroblasts, or amniocytes. Molecular genetic analysis is the standard method for confirmation of the diagnosis of Gaucher disease, and offers certain advantages over enzyme assays. All types of Gaucher disease can be detected during pregnancy through “amniocentesis” or “chorionic villus sampling.”^8^,^9^

### Treatment

The aim of the treatment of GD is reversal of organomegaly, prevention of complications and increase in quality of life. Severe organomegaly, high degree of cytopenia, event of minor bleeding due to thrombocytopenia, bone disease, liver enzyme elevation with sever organomegaly and presence of any organ involvement other than liver spleen, bone triad are indications of treatment Treatment is not effective for types 2 and 3 Gaucher disease because it cannot affect neurological manifestation because it cannot cross blood brain barrier Enzyme replacement for GD was FDA approved in 1991. ET with imiglucerase has become the standard of care for treatment of significantly symptomatic Gaucher disease type 1.^10^,^11^ Eliglustat (Cerdelga) is a new oral oral glucosylceramide synthase inhibitor indicated for the long-term treatment of adult patients with Gaucher disease type 1^12^, It is not affordable in Iraq under the financial hardship.

## METHODS

The studied group included 30 patients with Gaucher disease their ages range from (2-22 years) who were receiving enzyme therapy at pediatric gastroenterology department in Child welfare teaching hospital and Al-Imamenalkadhumen teaching hospital in Baghdad. Its prospective study started from July 2014 to January 2015, 17 patients diagnosed and were treated by ERT for one year before that date, and the others (thirteen patients) diagnosed during this period and started ERT, four patients for 6 months, six patients for three months and there are three patients were diagnosed at the end of 2014 and not completed 3 months of treatment, so they were not included in the enzyme assessment.

Diagnosis of these patients suspected by their clinical features and confirmed by demonstration of glucocerebrosidase deficiency. The test was done by taking blood samples from those with matched clinical features and put on special filter papers and sent by DHL either to a specialized metabolic laboratory in Hamburg University in Germany or to princess Haya biotechnology lab. In Jordan university of science & technology. This test became available in Iraq after 2010, so many of the patients had long period between their disease presentation and time of diagnosis. And also because of the shortage of filter papers for many months, made all the suspected cases waiting for quite some time before sent all together.

Genetic or molecular study was not done because there is no access to that test in our country. The type of the enzyme used in the treatment called Cerezyme (Genzyme Company) given every two weeks at a dose of (60 u/kg) in 200cc N/S iv infusion over 3 hours. The patients were examined before and after 3, 6 and12 months from the start of enzyme therapy by serial physical examination, complete blood count and abdominal u/s when available.

Anemia, thrombocytopenia and leukopenia were defined as follows: Hb values <10.5 g/dl were accepted as anemia for those 2-12 years old, and < 9.5 g/dl for those 6 months – 2 years old and < 10 for those below 6 months of age. Thrombocyte count <130X103/mm3 was defined as thrombocytopenia, and leukocyte count <4500/mm3 was defined as leucopenia.^13^

Hepatomegaly and splenomagaly were assessed by physical examination and abdominal ultrasound, Severity score index (SSI) was calculated for each patient by using Zimran’s scoring index.^14^ The data were reviewed, cleaned with double check entry into the computer using Statistical Package for Social Sciences (SPSS) version 20.

## RESULTS

This study included 18 males and 12 females. The mean age of the study group was 8.3 ± 6 years with range (2-22) years. Twenty eight patients diagnosed as type 1 while only 2 patients diagnosed as type 3. The highest percentage of the patients was from Baghdad. Positive consanguinity presented in 28 patients (93.3%). the most prevalent age group at presentation was (1 – 5) years, while the lowest age at presentation was >10 years (2 patients), with age range of (4 months – 14 years) years and mean of (3.1 ± 3.2) years.

Abdominal distension was the most common presenting symptoms (presented in 90% of the patients), bleeding tendency presented in more than half of the study group. Splenomegaly presented in all of the patients in our study while hepatomegaly presented in 26 patients (86%) Table-I.

**Table I:** Most common clinical and laboratory findings in the study group at presentation

Clinical laboratory findings	No. of patients affected (total no. 30)	Percentage
Splenomegaly	30	100%
Hepatomegaly	26	86.6%
Anemia	23	76.6%
Thrombocytopenia	19	63.3%
Skeletal radiographic findings	14	46.7%
Leucopenia	13	43.33%
Bone pain	12	40%

**Figure.1:**
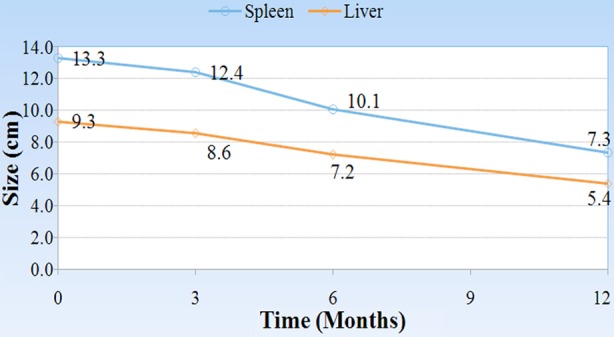
Changes in the size of Liver and Spleen in response to treatment regimen among patients with Gaucher disease.

Laboratory findings in the patient’s complete blood count were anemia which was presented in (76.6%), thrombocytopenia which was presented in (63.3%), and leukopenia presented in (43.33%). Table-I. there are seven patients who underwent splenectomy before starting ERT. Sixteen patients (53.3%) showed non radiological lesions while the rest (46.7%) showed different pictures of deformities, and the most prevalent one was Erlenmeyer deformity which presented in 33%. Bone marrow aspiration was found to be positive in 63.3%. After starting ERT we found that spleen and liver sizes highly decreased after 3, 6, 12 months from ERT with significant p-value 0.001 as we see in Figure 1. (Data records were not available in all of the patients).

**Figure.2:**
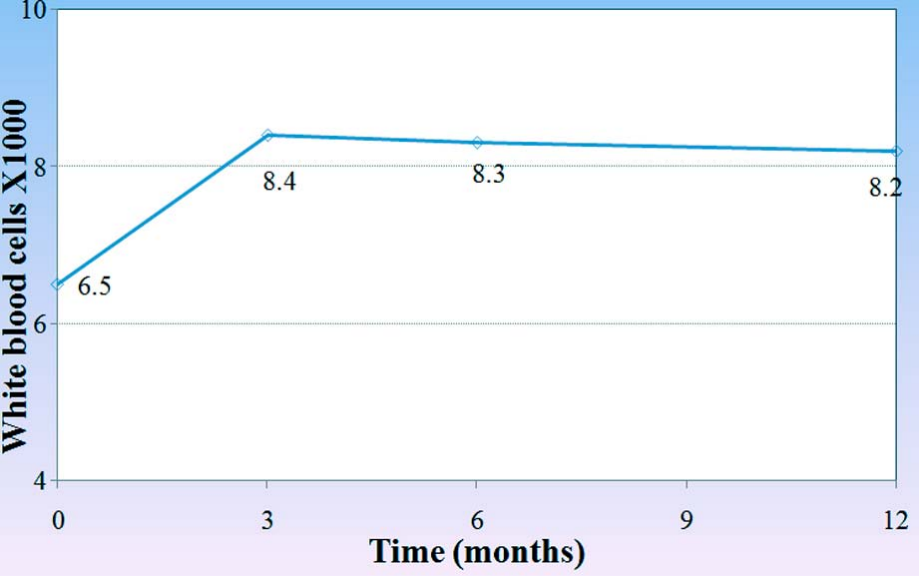
Changes in the mean count of white blood cells through-out the treatment course.

**Figure.3:**
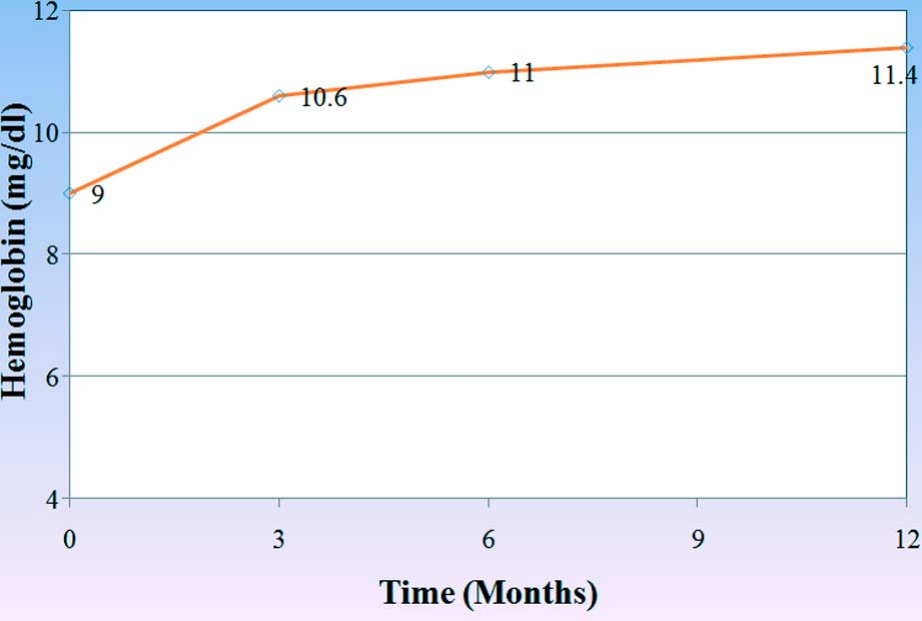
Changes in the mean level of hemoglobin through-out the treatment course.

**Figure.4:**
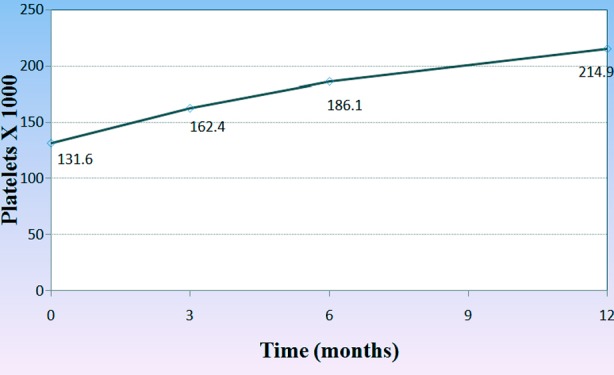
Changes in the mean count of platelets through-out the treatment course.

White blood cell count also increased with ERT after 6 and 12 months with significant p-value (0.031 and 0.042 respectively). Fig.2, on the other hand there was significant increase in hemoglobin level of the patients during the one year course of treatment. (3, 6, 12 months) (p<0.001, p=0.002 and p=0.001) respectively,Fig.3, and the platelets count also showed a significant increase after 3 months (p=0.032), 6 months (p<0.001) and one year (p=0.001) after treatment in comparison to its count before the beginning of the treatment Fig.4.

At presentation bone pain was present in 14/30 patients (60%) and after ERT and through-out the treatment course; it has been found that no significant deference in all of the patients, p-value (0.25). The Z-score of weight showed significant increase after 3, 6 months and one year of treatment (p=0.016, p=0.017 and p=0.001) respectively.

At time of diagnosis (14/24) patients height was below 5^th^ centile (60%), the height was not recorded in 6 patients. Also there was significant increase in the height after 1 year of treatment with p-value 0.011. The mean of severity scoring index (SSI) among the patients showed a highly significant decrease from 10.2±5.8 (range 3- 29) to 7.8±5.7 (range 2 – 27) after one year of treatment (p<0.001).

## DISCUSSION

The diagnosis of more than half of the patients was delayed for many years because of the lack of enzymatic test specific for Gaucher disease and unavailability of genetic study. The mean age at the start of treatment was 7.3 ± 5.2 years (range 1- 20 years), and the mean time between presentation and starting treatment was 3.7 years (range 3 months -14 years), this was long period before starting ERT because of the delayed in the diagnosis and unavailability of the treatment. Mean treatment period was 8.4 ± 4.4 months (range 3 – 12 months).

Disease presentation and clinical findings in our patients resembling that of V. Velmishi et al 2013.^15^ In this study 7 patients were spleenectomized before initiation of ERT. This is related to the long period between presentation of the disease and the start of ERT, which made many patients suffered from huge splenomegaly.

In comparison of complete blood count in patients who were splenectomized from those who were not, it was found that only white blood cells were higher among spleenectomized patients (p=0.002) while no significant differences were shown regarding hemoglobin and platelet counts (p value was 0.648 and 0.144 respectively). So it appeared that removal of the spleen in patients with Gaucher disease improved the white blood cells count only, and with the possible complications of such operation makes it unadvisable option (splenectomy).

This study also showed that there was no significant association in bone marrow infiltration with Gaucher cells between those who did spleenectomy and those who did not (p-value was >0.99). So from the above results it has been found that Gaucher disease affects spleen and bone marrow as independent variables.

In our country Gaucher enzyme replacement therapy started on May 2013 covered by ministry of health, many patients who were diagnosed as having Gaucher disease before that date but they couldn’t afford any treatment except one patient received treatment outside Iraq before that date. After ERT all patients showed decrease in the size of their spleen and liver after 3, 6 12 months of ERT, which mean a good response to the enzyme, and on the other hand, Hb level reached normal level in 67% of anemic patients after 6 months of treatment and the platelet count reached normal in 53.8% of the patients who presented with thrombocytopenia before treatment. While those patients who presented with bone pain showed on significant response after the whole one year of ERT. It is generally accepted that the improvement in bone disease requires a longer period than hematologic or organ-related complications of Gaucher disease. Organic and heamatological response in our patients are compatible with Arikan-ayyilidaz et al 2011.^16^ Mean weight and height z-score improved in all patients with ERT so linear growth accelerated after treatment.

The mean SSI in our study decreased from 10.2 to 7.8 after one year of treatment and it is compatible with many studies like Arikan-ayyilidaz et al. study using Zimran’s score, mean SSI decreased from 10.6 to 5.8 (period of treatment was more than our study). It’s worth to be mentioning that complications from ERT was not reported from any patient in the study group.

## CONCLUSION

The study showed that abdominal distension, splenomegaly, hepatomegaly and pacytopenia were most common presenting signs and symptoms; a high index of suspension is needed for early diagnosis and management. Complications from ERT were not reported in any patients in this study. Using ERT in treating patients with Gaucher disease was effective in the reversal of hematological complications, organomegaly and other changes in most of the patients, despite being started late. Splenectomy is not advisable in patients with Gaucher disease because its complication override the benefits.
